# Nutritional Attributes and Phenolic Composition of Flower and Bud of *Sophora japonica* L. and *Robinia pseudoacacia* L.

**DOI:** 10.3390/molecules27248932

**Published:** 2022-12-15

**Authors:** Jing Tian, Yuhong Gong, Jun Li

**Affiliations:** 1 Department of Life Science, Lvliang University, Lvliang 033001, China; 2 School of Food Science and Technology, Jiangnan University, 1800 Lihu Avenue, Wuxi 214122, China; 3 Institute of Food Processing Technology, Guizhou Academy of Agricultural Sciences, Guiyang 550006, China

**Keywords:** flavonoids, proximate composition, mineral elements, dietary fiber, amino acids, UPLC-QTOF-MS

## Abstract

*Sophora japonica* L. (SJL) and *Robinia pseudoacacia* L. (RPL) are widely cultivated in China. However, the utilization of their main by-products are limited due to a lack of comprehensive nutritional attributes. Herein, the proximate composition, mineral elements, fatty acids, amino acids, monosaccharides, and phenolics were analyzed to investigate the nutritional attributes of SJL and RPL. Dietary fiber was the main ingredient in SJL and RPL, followed by protein and lipids. The content of Fe in SJL and RPL was highest, especially in flowers of SJL, reaching about 1179.51 mg/kg. The total unsaturated fatty acids accounted for 89.67% of the bud of SJL. Meanwhile, the essential amino acids contents of the flower and bud of SJL and RPL accounted for 35.95–40.59% of total amino acids. The flower of SJL (373.75 mg/g) exhibited the most abundant monosaccharides. Meanwhile, the total phenolics and flavonoid contents in the buds of SJL and RPL were significantly higher than that of the flower, implying the buds possessed better biological activity. Moreover, the bud of SJL possessed the most abundant phenolics. The results provided a reference for the development of functional food derived from SJL and RPL.

## 1. Introduction

The buds of *Sophora japonica* L. (SJL), also named “Guohuai” in China, are known as *Flos Sophorae Immaturus*, a functional food widely used in China, Korea, and Japan. The functional properties of *Flos Sophorae Immaturus*, such as antioxidant properties, free radical scavenging, anti-inflammatory properties, hemostasis, blood sugar lowering, uric acid lowering, etc., were confirmed through extensive studies [1]. Xie et al. [2] determined 16 flavonoids in *Flos Sophorae Immaturus* through HPLC-DAD-ESI-MS/MS. Li et al. [3] found that polysaccharides from the buds of SJL protected HaCaT keratinocytes from ultraviolet-irradiation-induced skin injuries. Moreover, *Flos Sophorae Immaturus* contains various mineral elements, including K, Ca, Mg, Fe, Zn, Cu, etc. [1]. Therefore, *Flos Sophorae Immaturus* is mainly consumed as a functional tea. Unfortunately, the flowers of SJL are often ignored and considered lost application value, and no studies have focused on the difference between the main components of the flower and bud of SJL.

*Robinia pseudoacacia* L. (RPL) is also named “False acacia” or “Yanghuai” in China. Although RPL is considered an invasive species, it has been widely cultivated in China since 1970 due to its good soil and water conservation and ecological restoration effects [4]. The flower is the most abundant by-product of RPL and is usually used as a nectar source, as well as a vegetable in some parts of China, with a low utilization rate. Tian et al. [5] isolated five flavonoids from the ethanolic extract of the whole plant of RPL, including acacetin, secundiflorol I, mucronulatol, isomucronulatol, and isovestitol. Veitch et al. [6] determined 11 flavonoid glycosides in the leaves of RPL. Bratu et al. [7] indicated that the flower of RPL exhibited high antioxidant activity and that it induced significant necrosis and apoptosis of HeLa cells, providing an antitumor effect in vitro. Stankov et al. [8] found that the flower of RPL contained abundant concrete compounds (1.06%), including n-nonacosane, n-heptacosane, α-linolenic acid, n-pentacosane, palmitic acid, diisooctyl phthalate, etc. Therefore, the flower of RPL may be a promising food material.

Importantly, nutrient elements, including phenolics, fatty acids, mineral elements, and amino acids, were crucial for the development of functional foods. Liang et al. [9] determined the nutrition-related compounds of byproducts of sunflower seeds and revealed that the florets of sunflowers were rich sources of dietary fiber, Fe, and phenols. Hu et al. [10] investigated the phenolic composition and nutritional attributes of diaphragma juglandis fructus and walnut shells and revealed that these byproducts of walnut could be utilized for the development of health-beneficial functional foods, e.g., as phenolic antioxidants and weight loss. Meanwhile, high cellulose content in SJL and RPL exhibited promising application prospects in the preparation of hemicelluloses [11,12]. Additionally, polysaccharides also played a considerable role in the bioactivities of SJL and RPL [13]. Therefore, the analysis of basic components in SJL and RPL was important for the application of these materials. As the same plant species from the family Fabaceae, both the SJL and RPL gained tremendous attention of researchers. However, there is still an absence of nutritional composition and two groups of polyphenols in these two species. Thus, the objectives of our study were (1) to comparatively explor the nutritional properties of flowers and buds of SJL and RPL and (2) to identify monosaccharide and phenolic composition in the flowers and buds of SJL and RPL. It is foreseen that a clearer understanding of the primary functional components in SJL and RPL was obtained, which further contributed to the development of functional food derived from SJL and RPL.

## 2. Results and Discussion

### 2.1. Proximate Chemical Composition

The primary chemical content of SJL and RPL were shown in Table 1. Dietary fiber was the primary ingredient in both SJL and RPL, followed by protein and lipid. The soluble dietary fiber in the flowers of SJL (17.25 g/100 g) and RPL (19.29 g/100 g) were significantly higher than in the buds (*p* ≤ 0.05). Notably, the soluble dietary fiber contents of SJL and RPL were higher than barley (4.73–5.70 g/100 g) [14] and oat bran (8.9–14.2 g/100 g) [15]. A previous study showed that the consumption of soluble dietary fiber played a considerable role in alleviating cardiovascular disease and diabetes, reducing inflammation and cholesterol, and regulating gut microbiota [16]. Therefore, the abundant amount of soluble dietary fiber suggests SJL and RPL are beneficial to human health, which can be developed as functional food materials. Furthermore, the total phenolics and flavonoids contents in SJL were also obvious and significantly higher than that of RPL (*p* ≤ 0.05). Polyphenols are closely related to a variety of biological activities, including antioxidant, hypoglycemic, hypolipidemic, anti-bacterial, anti-hyperuricemia, anti-inflammatory, etc. [1]. Thus, SJL has a better utilization value compared to RPL. Meanwhile, the total phenolics and flavonoid contents in the buds of SJL and RPL were significantly higher than in the flowers (*p* ≤ 0.05), implying the buds possessed better biological activity. Interestingly, the polyphenols in SJL mainly consist of free phenolics, the amounts of which are significantly higher than those of bound phenolics (*p* ≤ 0.05), indicating that the phenolics compounds of SJL are more easily utilized. Moreover, SJL and RPL showed abundant contents of reducing sugar, ranging from 19.21 to 27.11 g/100 g, except for the buds of SJL.

### 2.2. Mineral Elements and Metals

The elemental compositions of SJL and RPL were shown in Table 1. The content of Fe in SJL and RPL was highest, especially in the flowers of SJL, reaching about 1179.51 mg/kg, which is similar to different types of tea [17]. Accumulating evidence has shown that Fe deficiency is closely related to fatigue, anemia, prematurity, and perinatal mortality [18]. Meanwhile, the recommended intake of Fe ions is 1, 10, 12, and 15 mg Fe kg/d for infants, children, and male and female adolescents, respectively, while the tolerable upper intake level is 45 mg/d [17]. Interestingly, the Fe contents in the flowers of SJL and RPL were higher than that of the buds, indicating the flowers might be a better Fe supplement for the human body. Furthermore, the Na contents in SJL and RPL were also in the leading position among all elements, which was beneficial to preserving the electrolyte environment in the human body. The contents of mineral compounds (Cu, Zn, Mn) in SJL and RPL were similar to herbal teas [19]. Cu, Zn, and Mn are essential components of plant enzymes and closely related to plant photosynthesis, respiration, and growth [20]. Notably, WHO has no allowable limits for mineral elements, as many of them are considered micronutrients. The contents of K, Ca, and Mg in SJL and RPL were lower than other elements, while heavy metals, such as Hg and Pb, were not detected. Moreover, a small amount of Se (0.029 mg/kg) was detected in the buds of RPL but not in other samples.

### 2.3. Fatty Acids

As shown in Table 2, twenty-seven fatty acids were measured in SJL and RPL, including twelve saturated fatty acids and fifteen unsaturated fatty acids. The total saturated fatty acid contents in the flower of SJL, flower, and bud of RPL were higher than unsaturated fatty acids, except in the bud of SJL. Palmitic acid was a primary saturated fatty acid in SJL and RPL, which accounted for 11.48%, 68.94%, 75.01%, and 63.12% in the flower of SJL, the bud of SJL, the flower of RPL, and the bud of RPL, respectively. Previous studies confirmed that palmitic acid exhibited analgesic, anti-inflammatory, antiviral, regulating lipid metabolism, and alleviating atherosclerosis properties [21]. Stearic acid, another major saturated fatty acid in SJL and RPL, has been shown to reduce low-density lipoprotein (LDL)-cholesterol [22]. The total unsaturated fatty acids accounted for 19.24%, 89.67%, 40.35%, and 40.81% of fatty acids in the flower of SJL, the bud of SJL, the flower of RPL, and the bud of RPL, respectively. The oleic content in the bud of SJL reached 1282.79 mg/kg, which is significantly higher than other samples (*p* ≤ 0.05). In contrast, the linoleic contents in the flower of SJL, flower and bud of RPL were dominant compared to oleic contents. Linoleic, a ω-6 series essential fatty acid, is beneficial to long-term glycemic control, cardiovascular risk, and insulin resistance [23]. Furthermore, linoleic can be metabolized into arachidonic acid, which is the precursor of eicosanoids, exhibiting proinflammatory or prothrombotic-vasoconstrictor properties [24]. The content of γ-linolenic (ω-6 family) was also abundant in the flower and bud of RPL, which is closely related to the decrease in blood concentrations of triacylglycerols, total cholesterol, and LDL [25]. SJL and RPL also contained a few polyunsaturated fatty acids (such as palmitoleic acid, eicosapentaenoic, and docosahexaenoic), which were beneficial to human health.

### 2.4. Amino Acids

As shown in Table 3, eleven nonessential amino acids (NEAA) and six essential amino acids (EAA) were identified in SJL and RPL. The EAA and NEAA contents in the buds of SJL and RPL were significantly higher than those in the flowers of SJL and RPL (*p* ≤ 0.05). The leucine content in the flower of SJL, the bud of SJL, the flower of RPL, and the bud of RPL reached 6.94 mg/g, 9.43 mg/g, 7.65 mg/g, and 7.67 mg/g, respectively, which is the most abundant of all EAA. Leucine, a branched-chain amino acid, showed a close relation to type 2 diabetes, insulin resistance, obesity, and nonmetabolic diseases. Notably, other branched-chain amino acids (valine and isoleucine) contents were also abundant in both SJL and RPL, which exhibited similar beneficial effects on humans [26]. Previous studies confirmed that branched-chain amino acids were the primary nitrogen sources for glutamine and alanine synthesis in muscle [9].

The most abundant NEAA in SJL and RPL was proline, with the contents ranging from 10.08 mg/g to 19.08 mg/g, respectively, while the bud of SJL possessed the highest content compared with other samples. Proline was also known as a primary precursor of extracellular collagens, against various potential harms (UV radiation, drought/salinity, heavy metals, reactive oxygen species), promotes beneficial tissue regeneration, and regulates cell signaling pathways [27]. The EAA contents of the flower of SJL, the bud of SJL, the flower of RPL, and the bud of RPL accounted for 40.59%, 35.95%, 35.95%, and 37.45% of total amino acids (TAA), respectively. The EAA/NEAA values of the flower of SJL, the bud of SJL, the flower of RPL, and the bud of RPL were 68.29, 56.13%, 56.14%, and 59.90%, respectively. Thus, the amino acid composition in the flower of SJL was more balanced than that in the bud of SJL according to the ideal protein standard for the human body suggested by FAO/WHO, which suggested 40% EAA/TAA and 60% EAA/NEAA as an appropriate amino acid composition. Interestingly, the opposite phenomenon occurred in the flower and bud of RPL.

To further evaluate the EAA composition of SJL and RPL, the amino acid scores of EAA (in 1 g of the sample protein) to the reference protein (in 1 g of the standard protein) of FAO/WHO were calculated. Lysine and threonine exhibited the lowest content in the flower and bud of SJL and RPL, implying these two amino acids were the first limiting amino acids. According to the standard amino acid composition recommended by FAO/WHO, an amino acid score close to 1 implies a more reasonable amino acid composition. As shown in Table 4, most amino acid scores of SJL and RPL were lower than 1, except methionine, cystine, and isoleucine, indicating a more reasonable composition of these three amino acids. Furthermore, the scores of the flower of SJL were higher than the bud of SJL, while the scores of the bud of RPL were higher than the flower of RPL, implying that the amino acid compositions of the flower of SJL and the bud of RPL were more balanced.

### 2.5. Monosaccharide Composition

The monosaccharide compositions of SJL and RPL were analyzed and shown in Table 5. The flower of SJL (373.75 mg/g) exhibited the most abundant monosaccharides, followed by the bud of SJL (198.92 mg/g), the flower of RPL (65.20 mg/g), and the bud of RPL (9.31 mg/g). The flowers of SJL and RPL showed the highest rhamnose contents, significantly higher than those of the buds. Rhamnose is a trace sugar that widely exists in plants and is often used as sweetener, flavor, and fragrance in the food industry [28]. The bud of SJL possessed the highest glucuronic acid content, significantly higher than that in the flower. However, glucuronic acid was not found in RPL. Previous studies confirmed that glucuronic acid could combine with a variety of harmful substances in the liver and provide a detoxification effect [29]. The arabinose content was abundant in both the flowers and buds of SJL and RPL. Arabinose can regulate intestinal peristalsis, control the accumulation of blood sugar and fat, and relieve a series of diseases such as diabetes and obesity [30]. Moreover, the xylose content was also abundant in these materials, which was widely used as xylitol material, low-calorie sweeteners, or food colorants in the food industry [31]. Furthermore, mannose, ribose, and galacturonic acid were also identified in SJL and RPL, indicating that hemicellulose was abundant in SJL and RPL. The results showed that SJL and RPL were important alternative renewable energy resources and provided a theoretical basis for future research and development.

### 2.6. Identification of Polyphenols

**Figure 1:**
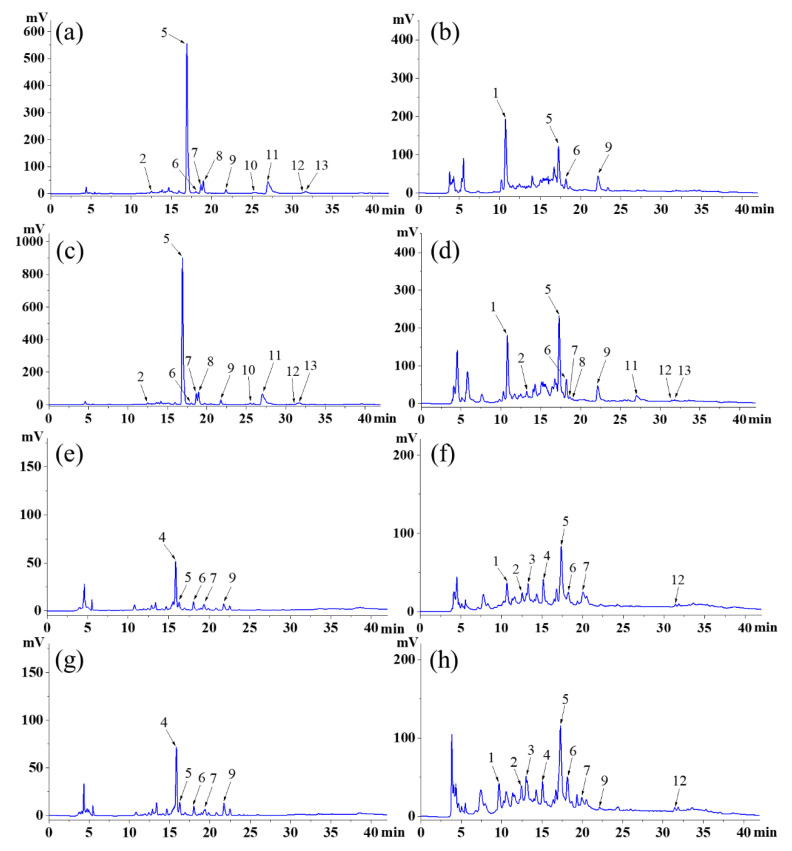
High-performance liquid chromatograms of (**a**) free phenolic compounds of the flower of *Sophora japonica* L.; (**b**) bound phenolic compounds of the flower of *Sophora japonica* L.; (**c**) free phenolic compounds of the bud of *Sophora japonica* L.; (**d**) bound phenolic compounds of the bud of *Sophora japonica* L.; (**e**) free phenolic compounds of the flower of *Robinia pseudoacacia* L.; (**f**) bound phenolic compounds of the flower of *Robinia pseudoacacia* L.; (**g**) free phenolic compounds of the bud of *Robinia pseudoacacia* L.; and (**h**) bound phenolic compounds of the bud of *Robinia pseudoacacia* L. The numbers of the peaks in this figure coincide with the compound numbers in Table 6. The chromatograms were recorded at 280 nm.

The chromatograms of phenolics compounds in SJL and RPL were shown in Figure 1a–h. Ten peaks were assigned to FP compounds (Figure 1a,c) in the flower and bud of SJL, while the FPs in the flower and bud of RPL had only five peaks. Moreover, the bud of SJL possessed the most abundant bound phenolics (10 peaks), followed by the bud of RPL (9 peaks), the flower of RPL (8 peaks), and the flower of SJL (4 peaks). Thus, SJL possessed more abundant free phenolics and RPL possessed more abundant bound phenolics. Compared to bound phenolics, free phenolics are easier to utilize. Therefore, SJL has greater advantages as a food material than RPL. Similar results showed that eight primary polyphenols were identified in Tartary buckwheat, consisting of six types of free polyphenols and six types of bound polyphenols [32]. Hu et al. [10] also found eleven types of free polyphenols and ten types of bound polyphenols in the diaphragma juglandis fructus. Therefore, SJL and RPL can be used as important sources of polyphenols.

The mass spectrometric data of identified phenolics are shown in Table 6. Compound 1 showed an [M-H]^−^ ion at *m/z* 153 and only existed in bound phenolics of SJL and RPL, so it was considered protocatechuic acid according to the previous studies [7,33]. Compound 2 showed an [M-H]^−^ ion at *m/z* 353, which was considered chlorogenic acid and also confirmed by other findings [7,33]. Compounds 3 and 4 only existed in RPL and revealed [M-H]^−^ ions at *m/z* 289 and 739, respectively. They were tentatively assigned as catechin and robinin according to the previous study [7]. Compounds 5, 6, and 7 existed in both SJL and RPL; they showed [M-H]^−^ ions at *m/z* 609, 463, and 593, so these compounds were tentatively assigned as rutin, hyperoside, and kaempferol-3-O-rutinoside, respectively [2,6]. Compounds 8, 10, 11, and 13 only existed in SJL and revealed [M-H]^−^ ions at *m/z* 623, 577, 301, and 315, respectively, so these compounds were assigned as narcissoside, sophorabioside, quercetin, and isorhamnetin, respectively based on the previous studies [33,34]. Compounds 9 and 12 showed [M-H]^−^ ions at *m/z* 447 and 285, which were assigned as quercitrin and kaempferol, respectively [6,35].

In general, the phenolics in SJL and RPL were mainly derivatives of quercetin, including rutin, kaempferol, isorhamnetin, hyperoside, quercitrin, etc. Interestingly, rutin was converted to quercetin and other derivatives by the rutin-degrading enzyme in SJL and RPL [36]. Previous studies also found that abundant derivatives of quercetin existed in SJL and RPL [33]. Therefore, the types of flavonoids in SJL and RPL are directly related to the planting environment and preservation methods. Moreover, accumulating evidence has shown that flavonoids exhibit better antihyperuricemia, antihyperglycemia, and antioxidant effects than phenolic acids [37,38]. Meanwhile, chemical reactions such as methylation, deglycosylation, and decarboxylation of flavonoids occurred during processing, which improved the conversion of these flavonoids with similar structures and further affected their bioactivities [39]. Therefore, studying appropriate processing methods in order to enhance the bioactivities of these flavonoids will be an interesting topic. Furthermore, SJL exhibited richer phenolic compositions and higher phenolic contents compared to RPL, implying that SJL possessed better application value in the food industry.

## 3. Materials and Methods

### 3.1. Materials and Chemicals

Fresh flowers and buds of SJL and RPL were obtained from indigenous trees (Zhaohe town, Nanyang, Henan Province, China), then dried in an oven (Binder, Neckarsulm, Germany) at 50 °C for 6 h. The dried samples were crushed to powder with a pulverizer (Wuyi Haina Electric Co., Ltd., Jinhua, China) and sieved through a 100-mesh screen. Rutin (≥98%), gallic acid (≥98%), α-amylase (10,000 u/mL), neutral protease (100 u/mg), and amyloglucosidase (100,000 u/mL) were obtained from Sinopharm Chemical Reagent Co., Ltd. (Shanghai, China).

### 3.2. Proximate Composition Analysis

Moisture, crude lipid, crude protein, ash, and dietary fiber contents were measured according to the AOAC (2005) methods. Total and reducing sugars were measured using the 3,5-dinitrosalicylic acid method [36]. The total phenolics and flavonoid contents were analyzed via the Folin–Ciocalteu method and aluminum chloride colorimetric method, respectively [37].

### 3.3. Mineral Elements and Metals Analysis

An inductively coupled plasma mass spectrometer (ICP-MS) instrument (Plasma Quant MS, Analytik Jena AG, Jena, Germany) was employed to determine the elements according to the method of Potorti et al. [40] with minor revisions. ICP-MS operated in no-gas mode for the isotopes Zn, Mn, Na, K, Ca, and Pb and in helium mode for Cu, Fe, Mg, Se, and Hg. The parameters were as follows: sample depth, 9 mm; generators power, 1500 W; nebulization chamber temperature, 2 ℃; nebulizer pump, 0.1 rps; extract lens, 1.5 V; collision gas flow rate, 4 mL/min.

### 3.4. Fatty Acid Analysis

The fatty acid composition was analyzed using a QP2010 Ultra GC-MS instrument (Shimadzu, Tokyo, Japan) equipped with a TG-5MS (30 m × 0.25 mm × 0.25 μm) chromatographic column [10]. Briefly, 100 mg of samples, 2 mL of 2% NaOH-methanol solution and 100 μL of methyl nonadecylate (interior label) were mixed. Then, the mixture was incubated at 40 °C for 20 min and extracted with 1 mL of n-hexane. The obtained supernatant was filled to 1 mL with n-hexane. The GC-MS parameters were as follows: injection volume, 1 μL; inlet temperature, 250 °C; the initial temperature was 80 ℃, then was increased to 230 °C at 10 ℃/min and held for 15 min; electron bombardment (EI) energy, 70 eV; ion source temperature, 230 °C; transfer line temperature 270 °C; solvent delay, 2 min; scanning range, 50–450 amu; scanning mode, total ion scanning.

### 3.5. Amino Acid Analysis

The amino acid composition was measured with a 1260 HPLC instrument (Agilent, Palo Alto, CA, USA) equipped with a DAD detector and a C18 column (4.6 × 250 mm × 5 μm) (Shiseido, Tokyo, Japan) [41]. First, samples were hydrolyzed with 6 mol/L HCl at 110 °C for 24 h. Then, the hydrolyzed samples were redissolved with 0.02 mol/L HCl solution after being drained in vacuum. Finally, the sample solution was mixed with 0.1 mol/L phenyl isothiocyanate acetonitrile solution and 1.0 mol/L triethylamine-acetonitrile solution. The determined samples were extracted by n-hexane. The liquid chromatography conditions were as follows: Solvent A, water mixed with 0.8% sodium acetate and 7% acetonitrile; Solvent B, acetonitrile mixed with 20% methanol and 20% water; the elution conditions were performed from 100% to 98% A in 5 min, from 98% to 95% A in 1 min, from 95% to 91% A in 8 min, from 91% to 79% A in 4 min, from 79% to 55% A in 14 min, from 55% to 45% A in 2 min, from 45% to 0% A in 4 min, from 0% to 0% A in 4 min, from 0% to 100% A in 3 min, from 100% to 100% A in 5 min; the detection wavelength, 254 nm; the flow rate, 1.0 mL/min; the column temperature, 30 °C; the injection volume, 10 μL.

### 3.6. Monosaccharide Compounds Analysis

The monosaccharide compounds were measured with the method of Wen et al. [42] with minor revisions. Briefly, the sample was hydrolyzed with 2 mol/L trifluoroacetic acid at 110 °C for 4 h, then adjusted the pH to 7 with 2 mol/L NaOH and centrifuged at 10,000 r/min for 10 min. Secondly, 0.4 mL of polysaccharide hydrolysate was mixed with 0.8 mL of 0.3 mol/L NaOH, and 0.5 mol/L 1-phenyl-3-methyl-5-pyra-zolone (PMP)-methanol solution, then derivatized at 70 °C for 60 min. Finally, 0.8 mL of 0.3 mol/L HCl was added to the mixture and extracted with chloroform five times. The supernatant was subjected to an HPLC instrument (Shimadzu, Kyoto, Japan) equipped with a UV/vis detector and a C18 column (5 μm, 4.6 mm × 250 mm i.d.). The liquid chromatography conditions were as follows: Solvent A, 15% acetonitrile with 0.05 mol/L phosphate buffer solution (KH_2_-PO_4_-NaOH, pH 7.1); Solvent B, 40% acetonitrile with 0.05 mol/L phosphate buffer solution (KH_2_PO_4_-NaOH, pH 7.1). The elution conditions were performed from 0% to 10% B in 10 min, from 10% to 30% B in 30 min, from 30% to 0% B in 5 min; the wavelength, 250 nm; the flow rate, 0.7 mL/min; the injection volume, 20 μL.

### 3.7. Polyphenols Preparation

Extraction of free phenolic (FP) compounds: 1.0 g of SJL or RPL powder was mixed with 30 mL of 70% (*v*/*v*) ethanol. The mixture was extracted on a magnetic stirrer at 25 °C for 40 min, and the speed was 300 r/min, then centrifuged at 5000 r/min for 20 min. The supernatants were evaporated with a rotary evaporator (RV8, IKA, Staufen, Germany) at 60 °C. Finally, the residues were redissolved in methanol and filled to 10 mL [43].

Extraction of bound phenolic (BP) compounds: The residues obtained from FP extraction were mixed with 40 mL of 3 mol/L NaOH solution. The mixture was incubated at 40 °C for 4 h then centrifuged at 6000 r/min for 20 min after adjusted pH to 2.0. The supernatant was extracted with ethyl acetate and evaporated at 50 °C using a rotary evaporator. Finally, the BP residues were redissolved with 2 mL of methanol [32].

### 3.8. UPLC-QTOF-MS Analysis of Polyphenols

FP and BP extracts were determined using a UPLC-QTOF-MS (Waters, Milford, MA, USA) equipped with a C18 column (2.1 × 150 mm, 1.7 μm) according to our previous study [31]. The mobile phase was comprised of acetonitrile (solvent A) and 0.1% acetic acid aqueous solution (solvent B) with a flow rate of 0.3 mL/min. The elution conditions were performed as follows: 0–5 min, 7–20% A; 5–24 min, 20–40% A; 24–28 min, 40–50% A; 28–40 min, 50–40% A; 40–42 min, 40–7% A. The injection volume was 10 μL and the chromatograms were recorded at 280 nm. The QTOF-MS parameters were as follows: negative mode; desolvation temperature, 400 °C; drying gas (N_2_) flow rate, 700 L/h; spray voltage, 3000 V; ion source temperature, 100 °C; mass spectra, 50 to 1500 Da.

### 3.9. Statistical Analysis

All experiments were performed in triplicate. Statistical analyses were performed with SPSS (version 22) and Origin (version 2021) software. The differences in data (*p* ≤ 0.05) were measured using one-way analysis of variance (one-way ANOVA).

## 4. Conclusions

In general, the proximate composition, mineral elements, fatty acids, amino acids, monosaccharides, and phenolic composition of SJL and RPL were clarified. The flowers and buds of SJL and RPL contained abundant phenolic compositions and mainly existed in a free state. Meanwhile, the soluble dietary fiber in the flowers of SJL (17.25 g/100 g) and RPL (19.29 g/100 g) was abundant. Moreover, Fe was the primary mineral element in SJL and RPL, especially in the flowers of SJL (1179.51 mg/kg). The total unsaturated fatty acids accounted for 19.24–40.81% in the flowers and buds of SJL and RPL. In particular, SJL and RPL also contained a few polyunsaturated fatty acids, such as palmitoleic acid, eicosapentaenoic acid, and docosahexaenoic acid. Furthermore, the amino acid composition of the flower of SJL and bud of RPL was more balanced according to the amino acid scores. Thus, richness in dietary fiber and mineral elements indicated that the flowers and buds of SJL and RPL could be applied to health-beneficial functional food development, e.g., regulating the gut ecosystem, functional food additives, weight regulation, and iron supplements. Likewise, abundant monosaccharide composition indicated that SJL and RPL were important alternate renewable energy resources. Thereafter, developing a beverage incorporating with micronized flowers and buds of SJL and RPL may be a promising strategy. Thus, the chemical composition variation of the flower and bud of SJL and RPL during the micronization process should be addressed in the future.

## Figures and Tables

**Table 1:** Proximate chemical composition (g/100 g dw), mineral and metal (mg/kg dw) contents of SJL and RPL.

Component	Flower of SJL	Bud of SJL	Flower of RPL	Bud of RPL
Moisture	5.74 ± 0.65 ^a^	5.70 ± 0.42 ^a^	6.48 ± 0.30 ^a^	6.27 ± 0.21 ^a^
Crude ash	10.38 ± 0.19 ^d^	7.24 ± 0.25 ^c^	5.30 ± 0.18 ^a^	5.92 ± 0.32 ^b^
Crude protein	11.65 ± 0.46 ^a^	16.82 ± 1.06 ^b^	21.30 ± 0.93 ^c^	21.65 ± 0.90 ^c^
Crude lipid	0.78 ± 0.07 ^a^	1.50 ± 0.10 ^c^	1.15 ± 0.04 ^b^	1.12 ± 0.04 ^b^
Reducing sugar	19.21 ± 0.34 ^b^	1.33 ± 0.23 ^a^	27.11 ± 0.54 ^c^	18.71 ± 0.60 ^b^
Total sugar	50.36 ± 2.58 ^b^	44.49 ± 1.70 ^a^	52.44 ± 2.48 ^b^	53.30 ± 3.37 ^b^
Soluble dietary fiber	17.25 ± 1.08 ^c^	7.91 ± 0.74 ^a^	19.29 ± 1.70 ^c^	12.23 ± 0.82 ^b^
Insoluble dietary fiber	27.92 ± 1.62 ^a^	33.58 ± 1.19 ^b^	27.29 ± 0.98 ^a^	37.06 ± 1.31 ^c^
Total dietary fiber	45.17 ± 2.69 ^ab^	41.49 ± 1.26 ^a^	46.57 ± 2.58 ^bc^	49.29 ± 1.08 ^c^
Free phenolics	8.05 ± 0.02 ^b^	11.41 ± 0.57 ^c^	0.58 ± 0.03 ^a^	0.67 ± 0.02 ^a^
Bound phenolics	0.10 ± 0.01 ^a^	0.13 ± 0.01 ^b^	0.11 ± 0.01 ^a^	0.18 ± 0.01 ^c^
Total phenolics	8.14 ± 0.02 ^b^	11.54 ± 0.57 ^c^	0.69 ± 0.03 ^a^	0.85 ± 0.02 ^a^
Free flavonoids	11.47 ± 0.10 ^b^	18.27 ± 1.39 ^c^	0.67 ± 0.01 ^a^	0.85 ± 0.01 ^a^
Bound flavonoids	0.12 ± 0.01 ^b^	0.16 ± 0.01 ^c^	0.10 ± 0.01 ^a^	0.19 ± 0.01 ^d^
Total flavonoids	11.59 ± 0.10 ^b^	18.43 ± 1.40 ^c^	0.77 ± 0.02 ^a^	1.04 ± 0.01 ^a^
Cu	11.94 ± 0.39 ^a^	16.25 ± 0.97 ^b^	11.98 ± 0.46 ^a^	12.47 ± 0.75 ^a^
Zn	25.22 ± 1.49 ^a^	29.92 ± 1.13 ^b^	31.98 ± 0.46 ^b^	36.47 ± 1.26 ^c^
Mn	46.89 ± 1.81 ^c^	35.58 ± 1.04 ^a^	39.98 ± 1.54 ^b^	49.13 ± 1.30 ^c^
Na	84.51 ± 1.47 ^c^	23.20 ± 1.87 ^a^	39.22 ± 2.05 ^b^	24.74 ± 1.07 ^a^
Fe	1179.51 ± 13.91 ^d^	53.20 ± 1.87 ^a^	175.56 ± 3.58 ^c^	133.41 ± 2.12 ^b^
K	2.21 ± 0.09 ^a^	2.97 ± 0.29 ^b^	2.29 ± 0.12 ^a^	2.31 ± 0.14 ^a^
Ca	0.75 ± 0.04 ^c^	0.60 ± 0.06 ^b^	0.15 ± 0.02 ^a^	0.21 ± 0.04 ^a^
Mg	0.31 ± 0.03 ^b^	0.39 ± 0.02 ^c^	0.12 ± 0.01 ^a^	0.16 ± 0.01 ^a^
Se	-	-	-	0.029 ± 0.003 ^a^
Hg/Pb	-	-	-	-

SJL: *Sophora japonica* L., RPL: *Robinia pseudoacacia* L. Different letters in the same row indicate significant differences in results (*p* ≤ 0.05).

**Table 2:** Fatty acid content of SJL and RPL (mg/kg dw).

Compound	Flower of SJL	Bud of SJL	Flower of RPL	Bud of RPL
Undecanoic acid (C11:0)	56.93 ± 0.87 ^d^	0.11 ± 0.02 ^a^	9.47 ± 0.34 ^b^	24.66 ± 0.47 ^c^
Lauric acid (C12:0)	78.80 ± 0.70 ^d^	0.13 ± 0.01 ^a^	5.37 ± 0.46 ^b^	8.93 ± 0.32 ^c^
Tridecanoic acid (C13:0)	56.73 ± 0.60 ^c^	-	13.70 ± 0.56 ^a^	29.25 ± 0.98 ^b^
Myristic acid (C14:0)	9.15 ± 0.32 ^b^	0.34 ± 0.04 ^a^	15.30 ± 0.38 ^d^	10.84 ± 0.27 ^c^
Pentadecanoic acid (C15:0)	60.67 ± 0.85 ^d^	0.12 ± 0.02 ^a^	14.59 ± 0.70 ^b^	20.82 ± 0.53 ^c^
Palmitic acid (C16:0)	81.47 ± 0.75 ^a^	128.07 ± 1.67 ^b^	626.03 ± 4.18 ^d^	456.77 ± 3.64 ^c^
Heptadecanoic acid (C17:0)	96.43 ± 0.93 ^d^	1.10 ± 0.05 ^a^	22.49 ± 1.03 ^b^	24.68 ± 0.72 ^c^
Stearic acid (C18:0)	65.93 ± 0.91 ^b^	55.37 ± 0.66 ^a^	82.42 ± 2.34 ^c^	81.07 ± 0.69 ^c^
Arachidic acid (C20:0)	-	0.28 ± 0.02 ^a^	-	-
Heneicosanoic acid (C21:0)	96.20 ± 0.61 ^d^	0.26 ± 0.01 ^a^	24.51 ± 0.66 ^b^	33.92 ± 0.72 ^c^
Behenic acid (C22:0)	3.69 ± 0.19 ^a^	-	-	-
Lignoceric acid (C24:0)	103.73 ± 1.70 ^c^	-	20.67 ± 0.52 ^a^	32.66 ± 0.88 ^b^
Total saturated	709.75 ± 1.56 ^b^	185.78 ± 2.34 ^a^	834.55 ± 7.39 ^d^	723.61 ± 4.24 ^c^
cis-10-Pentadecanoic acid (C15:1)	9.06 ± 0.24 ^b^	-	6.36 ± 0.20 ^a^	8.62 ± 0.41 ^b^
Palmitoleic acid (C16:1)	2.71 ± 0.30 ^b^	0.93 ± 0.03 ^a^	4.63 ± 0.21 ^c^	5.73 ± 0.27 ^d^
cis-10-Heptadecanoic acid (C17:1)	-	0.99 ± 0.03 ^a^	-	9.63 ± 0.42 ^b^
Oleic (C18:1)	33.36 ± 0.75 ^a^	1282.79 ± 9.29 ^c^	27.76 ± 0.59 ^a^	53.91 ± 0.37 ^b^
trans-6-Petroselenic (C18:1 T)	8.81 ± 0.35 ^c^	0.16 ± 0.03 ^a^	-	4.26 ± 0.28 ^b^
Linoleic (C18:2)	85.77 ± 1.23 ^a^	188.20 ± 1.48 ^b^	297.37 ± 3.17 ^d^	245.86 ± 3.62 ^c^
γ-Linolenic acid (C18:3 r)	17.58 ± 0.57 ^b^	2.04 ± 0.08 ^a^	150.16 ± 2.24 ^d^	134.86 ± 1.87 ^c^
cis-11-Eicosenoic acid (C20:1)	3.78 ± 0.25 ^a^	27.39 ± 0.54 ^c^	34.41 ± 0.67 ^d^	18.21 ± 0.67 ^b^
α-Linolenic acid (C18:3 a)	-	27.07 ± 0.59 ^b^	2.84 ± 0.11 ^a^	2.81 ± 0.14 ^a^
Eicosatrienoic (C20:3(2))	-	51.70 ± 1.12 ^c^	23.93 ± 0.93 ^b^	6.85 ± 0.29 ^a^
Arachidonic acid (C20:4)	3.35 ± 0.31 ^c^	1.96 ± 0.07 ^a^	1.84 ± 0.08 ^a^	2.39 ± 0.17 ^b^
cis-13,16-Docosadienoic acid(C22:2)	-	0.57 ± 0.04 ^a^	2.28 ± 0.34 ^b^	-
Eicosapentaenoic (C20:5)	4.71 ± 0.23 ^a^	27.53 ± 0.85 ^d^	12.91 ± 0.24 ^c^	5.72 ± 0.18 ^b^
Nervonic acid (C24:1)	-	0.12 ± 0.03 ^a^	-	-
Docosahexenoic acid (C22:6)	-	0.43 ± 0.05 ^a^	-	-
Total unsaturated	169.14 ± 2.44 ^a^	1611.89 ± 11.96 ^d^	564.48 ± 2.30 ^c^	498.85 ± 5.25 ^b^

SJL: *Sophora japonica* L., RPL: *Robinia pseudoacacia* L. Different letters in the same row indicate significant differences in results (*p* ≤ 0.05).

**Table 3:** Amino acid content of SJL and RPL (mg/g dw).

Amino Acids	Flower of SJL	Bud of SJL	Flower of RPL	Bud of RPL
Threonine (Thr)	1.75 ± 0.03 ^a^	2.17 ± 0.01 ^b^	2.58 ± 0.08 ^c^	2.98 ± 0.03 ^d^
Valine (Val)	5.64 ± 0.05 ^b^	5.16 ± 0.07 ^a^	5.84 ± 0.05 ^c^	6.61 ± 0.04 ^d^
Phenylalanine (Phe)	2.27 ± 0.06 ^a^	2.76 ± 0.07 ^b^	3.28 ± 0.04 ^c^	3.31 ± 0.02 ^c^
Isoleucine (Ile)	6.71 ± 0.06 ^a^	7.47 ± 0.02 ^b^	7.39 ± 0.07 ^b^	7.45 ± 0.03 ^b^
Leucine (Leu)	6.94 ± 0.10 ^a^	9.43 ± 0.08 ^c^	7.65 ± 0.06 ^b^	7.67 ± 0.05 ^b^
Lysine (Lys)	1.55 ± 0.07 ^a^	2.25 ± 0.03 ^b^	2.76 ± 0.04 ^c^	3.14 ± 0.07 ^d^
Total essential amino acids	24.85 ± 0.19 ^a^	29.23 ± 0.13 ^b^	29.50 ± 0.17 ^b^	31.17 ± 0.13 ^c^
Aspartic acid (Asp)	4.56 ± 0.09 ^a^	4.82 ± 0.07 ^b^	9.28 ± 0.08 ^d^	8.85 ± 0.05 ^c^
Serine (Ser)	2.10 ± 0.07 ^a^	2.50 ± 0.08 ^b^	3.15 ± 0.06 ^c^	3.49 ± 0.09 ^d^
Glutamate (Glu)	3.67 ± 0.06 ^a^	4.96 ± 0.08 ^b^	5.61 ± 0.10 ^c^	6.25 ± 0.06 ^d^
Glycine (Gly)	1.84 ± 0.05 ^a^	2.06 ± 0.07 ^b^	2.27 ± 0.03 ^c^	2.40 ± 0.03 ^d^
Alanine (Ala)	2.07 ± 0.04 ^a^	2.42 ± 0.05 ^b^	3.62 ± 0.06 ^d^	3.09 ± 0.07 ^c^
Cystine (Cys)	0.67 ± 0.04 ^c^	0.51 ± 0.02 ^a^	0.62 ± 0.02 ^b^	0.69 ± 0.02 ^c^
Methionine (Met)	6.19 ± 0.09 ^a^	8.81 ± 0.07 ^c^	7.29 ± 0.08 ^b^	6.20 ± 0.03 ^a^
Tyrosine (Tyr)	2.82 ± 0.06 ^a^	3.13 ± 0.04 ^b^	4.22 ± 0.04 ^c^	4.38 ± 0.06 ^d^
Histidine (His)	0.88 ± 0.05 ^a^	1.30 ± 0.02 ^b^	2.11 ± 0.03 ^c^	2.20 ± 0.05 ^d^
Arginine (Arg)	1.51 ± 0.03 ^a^	2.49 ± 0.03 ^b^	2.43 ± 0.06 ^b^	2.77 ± 0.09 ^c^
Proline (Pro)	10.08 ± 0.08 ^a^	19.08 ± 0.08 ^d^	11.96 ± 0.09 ^c^	11.73 ± 0.03 ^b^
Total nonessential amino acids	36.39 ± 0.28 ^a^	52.08 ± 0.50 ^b^	52.55± 0.09 ^b^	52.04 ± 0.16 ^b^
Total amino acids	61.24 ± 0.22 ^a^	81.31 ± 0.63 ^b^	82.06 ± 0.25 ^c^	83.22 ± 0.24 ^d^

SJL: *Sophora japonica* L., RPL: *Robinia pseudoacacia* L. Different letters in the same row indicate significant differences in results (*p* ≤ 0.05).

**Table 4:** Essential amino acid composition of compared to the FAO/WHO pattern. (mg/100 g protein dw).

Amino Acids	FAO/WHO Pattern	Flower of SJL	Bud of SJL	Flower of RPL	Bud of RPL
Threonine	40	0.38	0.32	0.30	0.34
Valine	50	0.97	0.61	0.55	0.61
Methionine + Cystine	35	1.69	1.57	1.06	0.91
Isoleucine	40	1.44	1.10	0.87	0.86
Leucine	70	0.85	0.79	0.51	0.50
Phenylalanine + Tyrosine	60	0.73	0.58	0.59	0.59
Lysine	55	0.24	0.24	0.24	0.26

SJL: *Sophora japonica* L., RPL: *Robinia pseudoacacia* L.

**Table 5:** Identification of monosaccharide compounds of SJL and RPL (mg/g dw).

Compound	Flower of SJL	Bud of SJL	Flower of RPL	Bud of RPL
Mannose	15.38 ± 1.35 ^b^	-	8.78 ± 0.31 ^a^	-
Ribose	23.79 ± 1.98 ^c^	4.46 ± 0.43 ^b^	5.35 ± 0.61 ^b^	0.90 ± 0.06 ^a^
Rhamnose	105.18 ± 2.59 ^d^	4.63 ± 0.48 ^b^	23.24 ± 0.98 ^c^	1.09 ± 0.23 ^a^
Glucuronic acid	5.38 ± 0.35 ^a^	99.87 ± 1.50 ^b^	-	-
Galacturonic acid	74.99 ± 1.35 ^d^	5.24 ± 0.24 ^b^	15.96 ± 0.21 ^c^	0.36 ± 0.04 ^a^
Xylose	102.39 ± 3.33 ^c^	32.45 ± 1.98 ^b^	5.39 ± 0.81 ^a^	1.68 ± 0.20 ^a^
Arabinose	46.65 ± 0.90 ^b^	52.27 ± 4.47 ^c^	6.49 ± 0.18 ^a^	5.28 ± 0.72 ^a^
Total content	373.75 ± 3.64 ^d^	198.92 ± 7.85 ^c^	65.20 ± 0.21 ^b^	9.31 ± 0.74 ^a^

SJL: *Sophora japonica* L., RPL: *Robinia pseudoacacia* L. Different letters in the same row indicate significant differences in results (*p* ≤ 0.05).

**Table 6:** Mass spectrometric data, and identification of polyphenols extracted from SJL and RPL.

Peak	UV λ/nm	Compounds	[M-H]^−^ *m*/*z*	Flower of SJL		Bud of SJL		Flower of RPL		Bud of RPL	
				fp	bp	fp	bp	fp	bp	fp	bp
1	213, 259	Protocatechuic acid	153	−	+	−	+	−	+	−	+
2	211, 289	Chlorogenic acid	353	+	−	+	+	−	+	−	+
3	239, 263	Catechin	289	−	−	−	−	−	+	−	+
4	265, 344	Robinin	739	−	−	−	−	+	+	+	+
5	256, 356	Rutin	609	+	+	+	+	+	+	+	+
6	253, 367	Hyperoside	463	+	+	+	+	+	+	+	+
7	264, 342	Kaempferol-3-O-rutinoside	593	+	−	+	+	+	+	+	+
8	258, 342	Narcissoside	623	+	−	+	+	−	−	−	−
9	258	Quercitrin	447	+	+	+	+	+	−	+	+
10	262, 313	Sophorabioside	577	+	−	+	−	−	−	−	−
11	255, 372	Quercetin	301	+	−	+	+	−	−	−	−
12	261, 367	Kaempferol	285	+	−	+	+	−	+	−	+
13	253, 368	Isorhamnetin	315	+	−	+	+	−	−	−	−

“+” indicates that it was detected in this sample, “−” indicates that it was not detected in this sample, SJL: *Sophora japonica* L., RPL: *Robinia pseudoacacia* L.

## Data Availability

Data are contained within the article.

## Sample Availability

Samples of the compounds are not available from the authors.
